# Differences in negative predictive value of prostate MRI based in men with suspected or known cancer

**DOI:** 10.1590/0100-3984.2018.0126

**Published:** 2019

**Authors:** Armonde A. Baghdanian, Yoon-Jin Kim, Arthur H. Baghdanian, Hao N. Nguyen, Katsuto Shinohara, Antonio C. Westphalen

**Affiliations:** 1 University of California, San Francisco, Department of Radiology and Biomedical Imaging, San Francisco, CA, USA.; 2 University of California, San Francisco, Department of Urology, San Francisco, CA, USA.

**Keywords:** Prostate cancer, Active surveillance, Magnetic resonance imaging, Prostate biopsy, Multiparametric MRI, Câncer de próstata, Vigilância ativa, Ressonância magnética, Biópsia

## Abstract

**Objective:**

To compare the negative predictive value (NPV) of multiparametric MRI for Gleason score (GS) ≥ 3+4 cancer and evaluate predictors of these tumors in men with suspected disease and under active surveillance (AS).

**Materials and Methods:**

This retrospective study included 38 men with suspected prostate cancer and 38 under AS with scans assigned PI-RADS v2 scores 1 or 2 between May 2016 and September 2017. Biopsy results were no cancer, GS = 3+3, or GS ≥ 3+4. Pre-MRI PSA, gland volume, and PSA density were recorded. Chi-square, equality of proportions, and logistic regressions were used to analyze the data.

**Results:**

Intermediate to high-grade cancer was found in 12.8% (95% CI = 2.3-23.3) and 35.9% (95% CI = 20.8-50.9) of men with suspected cancer, and under AS (*p* = 0.02), respectively. The NPV for GS ≥ 3+4 were 87.2% (suspected cancer; 76.7-97.7) and 64.1% (AS; 49.0-79.2). In neither group PSA significantly predicted cancer grade (*p* = 0.75 and 0.63). Although it did not reach conventional statistical significance, PSA density was a good predictor of cancer grade in men with suspected disease (*p* = 0.06), but not under AS (*p* = 0.62).

**Conclusion:**

The NPV of multiparametric MRI for GS ≥ 3+4 is higher in men with suspected prostate cancer than in men under AS. PSA density ≤ 0.15 improved the prediction of intermediate to high-grade disease in patients without known cancer.

## Abstract (pt)

**Objetivo:**

Comparar o valor preditivo negativo (VPN) da RM multiparamétrica da próstata para o diagnóstico de tumores escore de Gleason (EG) ≥ 3+4 e avaliar os preditores desses tumores em homens com suspeita de doença e nos sob vigilância ativa (VA).

**Materiais e Métodos:**

Este estudo retrospectivo incluiu 38 homens com suspeita de câncer de próstata e 38 em VA com RM, aos quais foram atribuídos escores PI-RADS v2 1 ou 2 entre maio de 2016 e setembro de 2017. Os resultados da biópsia foram ausência de câncer, câncer EG = 3+3 ou câncer EG ≥ 3+4. PSA pré-RM, volume da glândula e densidade de PSA foram anotados. Qui-quadrado, igualdade de proporções e regressões logísticas foram utilizados para analisar os dados.

**Resultados:**

Câncer de grau intermediário a alto grau foi encontrado em 12,8% (IC 95% = 2,3–23,3) e 35,9% (IC 95% = 20,8–50,9) dos homens com suspeita de câncer e nos sob VA (p = 0,02), respectivamente. O VPN para GS ≥ 3+4 foi 87,2% (suspeita de câncer; IC 95% = 76,7–97,7) e 64,1% (VA; IC 95% = 49,0–79,2). Em nenhum dos grupos o PSA previu significativamente o grau de câncer (p = 0,75 e 0,63. Embora não tenha alcançado o limiar de significância estatística usual, a densidade de PSA foi um bom preditor de grau de câncer em homens com suspeita de doença (p = 0,06), mas não sob VA (p = 0,62).

**Conclusão:**

O VPN da RM multiparamétrica para GS ≥ 3+4 é maior em homens com suspeita de câncer de próstata do que em homens sob VA. Uma densidade de PSA ≤ 0,15 melhorou a previsão de doença de grau intermediário a alto grau em pacientes sem diagnóstico prévio de câncer.

## INTRODUCTION

Recently, multiparametric magnetic resonance imaging (mpMRI) has become the imaging modality of choice for the assessment of men with known or suspected prostate cancer. In particular, it has been utilized to identify regions within the gland that are most likely to harbor intermediate to high-grade disease, direct biopsies, and, therefore, impact management.

Although several authors have reported very high negative predictive values (NPVs) of mpMRI, this still remains a matter of contention and debate among specialists involved in the care of prostate cancer. For example, Lee et al. reported a NPV of only 45.2%^(^^1^^)^, while Otti et al. reported 85%^(^^2^^)^. Tran et al. reported that systematic biopsy detected tumors with Gleason score (GS) ≥ 4+3 in 9% of men with negative MRI-ultrasound fusion biopsies^(^^3^^)^, although Garcia-Reyes et al. reported that only 1.2% of samples that were obtained from sites without lesions on mpMRI represented GS 4+3 or higher^(^^4^^)^.

Several potential reasons can be given to explain the discrepant findings amongst the above-mentioned studies, but one potential confounder is the variability in disease prevalence from study to study. As the NPV of any test is dependent on the prevalence of disease, tests utilized in populations in which the event is rare will always have higher NPVs than tests applied to populations in which the disease is common. The study by Lee et al.^(^^1^^)^, for example, was conducted in men who underwent radical prostatectomy, while the study of Garcia-Reyes et al.^(^^4^^)^ was of a population with suspected cancer or under active surveillance (AS), in whom the prevalence of high-grade disease is expected to be much lower.

In the present study we compared the NPV of mpMRI for intermediate or high-grade prostate cancer and evaluated predictors of these tumors in men with suspected disease and patients under AS.

## MATERIALS AND METHODS

This was a HIPPA-compliant, Institutional Review Board-approved with a waiver of informed consent retrospective cohort study.

### Patient population

A single author (AAB) used Nuance mPower Clinical Analytics (Nuance Communications, Inc.; Burlington, MA, USA) to search all prostate mpMRI reports with the words “PI-RADS v2 score” between May 2016 and September 2017 to identify all consecutive scans with PI-RADS scores 1 or 2. This range of time was chosen as all patients undergoing a prostate mpMRI were graded based on the Prostate Imaging Reporting and Data System (PI-RADS) version 2 grading system at our institution and had obtained a biopsy after mpMRI completion.

The detailed inclusion criteria for the study were as follows: complete prostate mpMRI examination performed at our institution; MRI initial interpretation performed using the PI-RADS grading system with a final score of 1 or 2; the indication for mpMRI was either for suspected prostate cancer or monitoring of patients under AS; systematic transrectal ultrasound (TRUS)-guided biopsy of the prostate within 12 months after mpMRI examination.

TRUS-guided biopsy information was derived from the electronic medical record. Exclusion criteria included incomplete or non-retrievable pathology results, lack of a serum prostate-specific antigen (PSA), lack of a calculated prostate volume, and an incomplete TRUS-guided biopsy.

### Data collection

Once our patient cohort was established, a second author (YJK) reviewed the electronic medical record to obtain patient demographic information, history and grade of prior untreated prostate cancer, and pre-mpMRI serum PSA values. Prostate volume was obtained from the mpMRI examination reports and was used to calculate each patient’s mean PSA density (PSAD). Furthermore, post-mpMRI biopsy results were classified as no cancer, low-grade cancer, or high-grade cancer. Patients were designated as having low-grade cancer if the highest GS diagnosed on TRUS-guided biopsy was 3+3 (International Society of Urological Pathology [ISUP] group 1) and as having intermediate to high-grade cancer if the highest GS was 3+4 or higher (ISUP groups 2-5). The presence of known cancer prior to TRUS-guided biopsy was noted. In patients with known cancer prior to biopsy, the highest GS, which could be derived from the procedure performed before or after mpMRI, was considered as the outcome. We made this option because underestimation of the GS by the post-mpMRI TRUS-guided biopsy is more likely than a real decrease in GS.

### MRI imaging technique

mpMRI studies were performed with a 3.0 T whole-body MR scanner (GE Healthcare; Waukesha, WI, USA). All patients were imaged in a supine position using a body coil for excitation, and a pelvic phased-array coil (GE Healthcare; Waukesha, WI, USA) and an endorectal coil (Medrad; Pittsburgh, PA, USA) for signal reception. After a three-dimensional localizer scan, axial T2-weighted and diffusion-weighted MR images were obtained. Dynamically contrast enhanced images were also acquired. Protocol details are given in Table 1.

**Table 1:** Acquisition parameters for the mpMRI of the prostate with endorectal coll.

Series	PSD	Scan plane	TR (ms)	TE (ms)	Slice/gap (mm)	FOV (mm)	Acquisition matrix	NEX	Sequence specific
Scout	FSE	3-plane	867	83	5/1.5	400 × 400	256 × 192	1	—
T1	FGRE	Axial	5.06	2.46	4.2/0	240 × 240	192 × 128	1	3D
T2	FSE	Oblique axial	5000	96	3/0	180 × 180	256 × 256	3	2D
T2	FSE/CUBE	Oblique axial	2400	142.5	1.6/0	180 × 180	256 × 224	1	3D reformatting recommended
DWI mid	ss-EPI	Oblique axial	4725	Min	3/0	180 × 180	128 × 64	6	b = 600 s/mm^2^; rFOV recommended
DWI high	ss-EPI	Oblique axial	4725	Min	3/0	260 × 260	128 × 64	7	b = 1350 s/mm^2^
DCE	3D SPGR	Oblique axial	Min	Min	3/0	260 × 260	192 × 128	1	Temporal resolution = 10 s

### Image interpretation

All scans were originally interpreted by one of 13 board-certified abdominal imaging fellowship-trained radiologists with at least three years of experience reading prostate mpMRI examinations. Radiologists were aware of all clinical information at the time of interpretation. Examinations were evaluated on a dedicated workstation and software (DynaCAD Prostate; Invivo Corporation, Gainesville, FL, USA). Gland segmentation was performed on the software by the radiologist and used to calculate the gland volume. Each mpMRI examination was evaluated using the PI-RADS official manual published in January of 2015^(^^5^^)^. The interpreting radiologist was provided with a standardized dictation template used for grading the examination based on the PI-RADS guidelines. PI-RADS score of 1 was assigned when no abnormalities were seen in the peripheral zone (PZ) or transition zone (TZ). A PI-RADS score of 2 was assigned: a) when no abnormalities were seen in the PZ, but benign prostatic hyperplasia was present in the TZ; b) when linear or wedge-shaped foci of low T2 signal intensity were present in the PZ, with benign prostatic hyperplasia present or absent on the TZ; and c) when indistinct diffuse mild low signal intensity was seen in the PZ, with benign prostatic hyperplasia present or absent on the TZ.

### TRUS-guided biopsies

Two urologists performed all TRUS-guided biopsies as part of clinical care (49/78 cases-KS, years of overall and fusion biopsy experience = 34 and 6; and 29/78 cases-HNN, years of experience = 4 and 4). Real-time cine and still images were obtained in two planes using high-resolution B-mode ultrasound with a 6-9 Hz TRUS probe (Philips Healthcare; Amsterdam, The Netherlands). A focus with lower echogenicity than the adjacent tissue was considered a positive finding on TRUS. Color Doppler was utilized, but the presence of increased vascularity was not mandatory to proceed with a biopsy. Depending on the size of the lesion identified on ultrasound, one or two samples were taken from the center and from the periphery of the lesion. These were followed by a 14-core extended-sextant systematic biopsy of the right and left anterior TZ, and medial and lateral locations of the PZ at the apex, midgland, and base. Targeted and systematic biopsies were performed by the same urologist in one session.

### Statistical analysis

Chi-square was used to compare the distribution of GS between men with suspected prostate cancer or under AS. Similarly, the equality of proportions test was used to compare the proportion of men in these two groups who were diagnosed with low and intermediate to high-grade prostate cancer after the negative mpMRI. We also used logistic regressions to generate 95% CI and compare the proportion of cancer diagnosis, and to determine if baseline PSA and baseline PSAD were predictors of intermediate to high-grade prostate cancer in each one of these two subpopulations. All analyses were done using Stata 13 (StataCorp LP; College Station, TX, USA). A 5% level of confidence was considered significant for all tests.

## RESULTS

A total of 78 patients met our inclusion criteria: 39 (50%) were suspected to have prostate cancer (median age, 62-years; interquartile range [IQR], 55-67) and 39 (50%) were under AS (median age, 67-years; IQR, 60-71). Men under AS had pre-mpMRI GS 3+3 (n = 31), 3+4 (n = 6), and 4+3 (n = 2) cancers. No patients were excluded from our study.

mpMRI scans were ordered as part of clinical care. The mean time interval between mpMRI and TRUS-guided biopsy was 63 days (standard deviation [SD] = 70.2); 90% of men had a biopsy done within 168 days of mpMRI.

On biopsies performed after mpMRI, low-grade cancer was diagnosed in 23.1% (9/39; 95% CI = 9.9-36.3) of men with suspected prostate cancer in comparison to 64.1% (25/39; 95% CI = 49.0-79.2) of men under AS (*p* < 0.001). Conversely, intermediate to high-grade cancer was found in 12.8% (5/39; 95% CI = 2.3-23.3) of patients suspected to have prostate cancer and in 35.9% (14/39; 95% CI = 20.8-50.9) of men under AS (*p* = 0.02).

The NPV for intermediate to high-grade cancer were 87.2% (suspected cancer group; 76.7-97.7) and 64.1% (AS group; 49.0-79.2). Table 2 summarizes the distribution of GS in our population.

**Table 2:** Post-MRI biopsy results per baseline patient group.

	Gleason score						
	Negative	3+3 (ISUP 1)	3+4 (ISUP 2)	4+3 (ISUP 3)	4+4 (ISUP 4)	4+5 or higher (ISUP 5)	Total
Suspected PCa	25	9	2	3	0	0	39
AS	0	25	8	5	1	0	39
Total	25	34	10	8	1	0	78

PCa, prostate cancer.

The mean PSA (ng/mL) of men with suspected prostate cancer and diagnosed with (a) intermediate to high-grade prostate cancer was 6.2 (SD = 2.1); (b) low-grade prostate cancer was 11.9 (SD = 12.6); and (c) no cancer was 10.0 (SD = 5.9). The mean PSA of men under AS and diagnosed (a) with intermediate to high-grade prostate cancer was 5.5 (SD = 4.6); and (b) low-grade prostate cancer was 6.1 (SD = 3.3). Serum PSA was neither a significant predictor of prostate cancer grade in patients suspected to have prostate cancer (*p* = 0.75), nor in patients under AS (*p* = 0.63).

The mean gland volume of men with suspected prostate cancer and under AS were 67.5 mL (SD = 38.2) and 59.3 mL (SD = 32.8) (*p* = 0.32).

The mean PSAD (ng/mL/mL) of men with suspected prostate cancer and diagnosed with a) intermediate to high-grade prostate cancer was 0.24 (SD = 0.16); b) low-grade prostate cancer was 0.20 (SD = 0.12); and c) no cancer was 0.15 (SD = 0.09). The mean PSAD of men under AS and diagnosed a) with intermediate to high-grade prostate cancer was 0.12 (SD = 0.08); and b) low-grade prostate cancer was 0.11 (SD = 0.07). PSAD nearly reached the standard definition of a significant predictor of prostate cancer grade in men with suspected disease (*p* = 0.06), but not in men under AS (*p* = 0.62).

**Figure 1:**
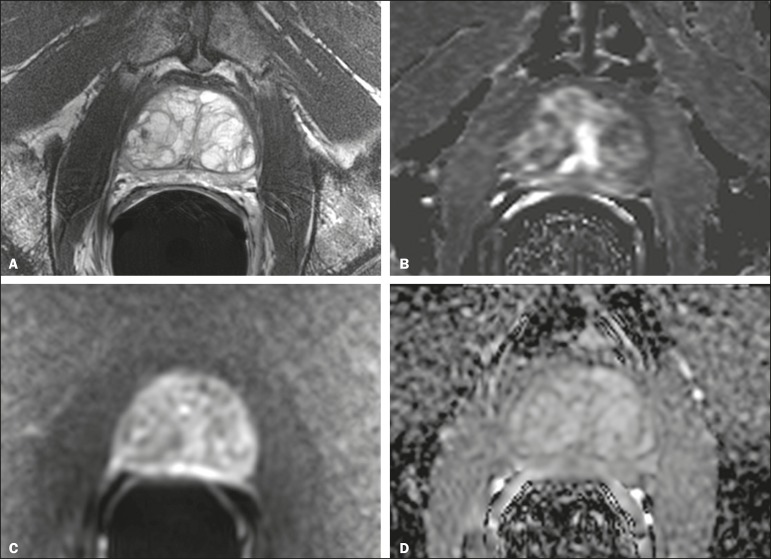
67-year-old man with suspected prostate cancer and PSAD of 0.09 (baseline total serum PSA = 4.4 ng/mL, and gland volume = 49 mL). No suspicious findings were seen on MRI. Representative images are shown—**A**: T2-weighted image; **B**: Dynamic contrast enhanced MRI parametric map; **C**: High b-value diffusion-weighted MRI; **D**: apparent diffusion coefficient map. Systematic biopsy diagnosed GS 4+3 cancer in the anterior left midgland (5% of the total length of cores, 1 of 21 mm).

**Figure 2:**
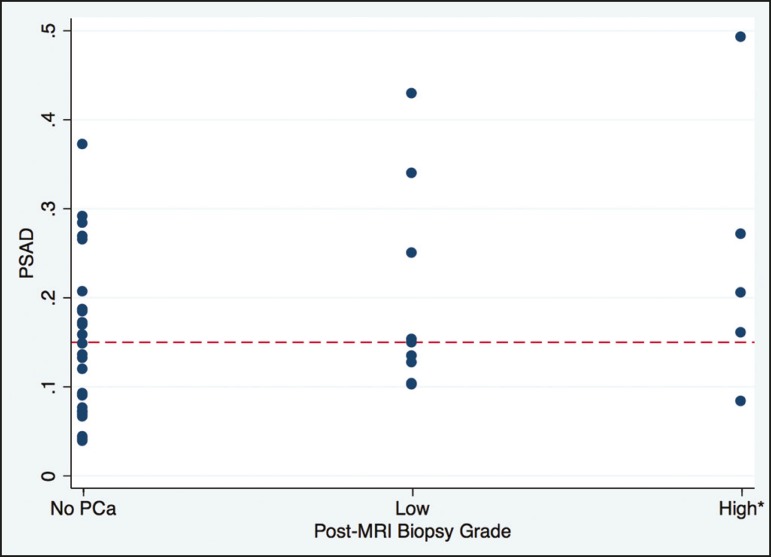
Distribution of PSAD by prostate cancer grade on post-MRI biopsy, where low grade prostate cancer is defined as GS 3+3 (ISUP group 1) and high-grade cancer is defined as GS ≥ 3+4 (ISUP groups 2-5).

A commonly used PSAD threshold for clinical management is 0.15^(^^6^^)^. Only one patient with suspected prostate cancer and a PSAD ≤ 0.15 had intermediate to high-grade prostate cancer on biopsy (1/20; 5%). This patient had a PSAD = 0.09 and a GS 4+3 tumor, with high-grade disease seen in 5% of the total length of cores (1 of 21 mm) (Figure 1). In other words, the NPV of a negative mpMRI in association with a PSAD < 0.15 was 95% (95% CI = 57.2-100). Additionally, only 4 men (4/18; 22%) had low-grade prostate cancer in association with a PSAD > 0.15 These results are illustrated in Figure 2. In men under AS, however, 9 of 27 men had intermediate to high-grade prostate cancer in association with a PSAD < 0.15, for a NPV of 67% (95% CI = 39.5-100). Low-grade prostate cancer was seen in 7 of 12 men under AS (58%).

## DISCUSSION

Aggressive management of patients suspected of having prostate cancer and of patients under AS can lead to adverse events in diagnosis with invasive biopsy techniques or overtreatment of tumors that are considered indolent. With this understanding, more patients undergo mpMRI to help determine the presence of intermediate to high-grade tumors that can be diagnosed using TRUS-MRI fusion biopsy. Often times though, mpMRI does not identify any suspicious lesions in these populations. The next step in the management of these men is typically a systematic TRUS-guided biopsy. The results of this study suggest that men with suspected prostate cancer, a negative mpMRI scan, and a PSAD < 0.15 may avoid an immediate biopsy.

PROMIS, which investigated the value of mpMRI to triage men with suspected prostate cancer based on elevated PSA, estimated based on transperineal mapping the NPV of mpMRI for the detection of prostate cancer GS ≥ 3+4 in 76% (95% CI = 69-82)^(^^7^^)^. Our results are aligned with those of PROMIS when we consider men with suspected prostate cancer, but not if they are under AS. The NPV of mpMRI for intermediate or high-grade disease was significantly lower in men under AS than in those with suspected cancer. In this population, therefore, a negative mpMRI, irrespective of PSA and PSAD levels, may not be sufficient to exclude disease upgrading and a systematic biopsy should be considered.

An et al. attempted to identify predictors of high-grade prostate cancer in the setting of a negative mpMRI, but none of the variables they studied (age, race, clinical stage, prostate volume, and PSA) were helpful^(^^8^^)^. Similarly, serum PSA level was not a predictor of intermediate to high-grade prostate cancers in either group of patients in our study. Men without cancer and with low-grade disease had higher serum PSA than those with intermediate to high-grade cancer. While we can only speculate on the reasons for it, the result is consistent with other prior studies that have demonstrated that moderately elevated PSA values (4.0 to 10.0 ng/mL) lack specificity and that 75% of biopsies in these patients are negative and unnecessary. Likewise, the use of age-adjusted PSA cut-offs has been shown to miss nearly 20% of cancers of men in their 60s and nearly 60% of cancers of men in their 70s^(^^9^^,^^10^^)^.

PSAD, however, has been shown to be more sensitive and specific than serum PSA for the detection of prostate cancer^(^^11^^-^^13^^)^. Washino et al. demonstrated that biopsies performed in patients suspected of having prostate cancer with a PI-RADS score ≤ 3 and a PSAD < 0.15 ng/mL/mL yielded no clinically significant prostate cancer^(^^14^^)^. Our results corroborate their findings. PSAD was an independent predictor of a positive biopsy outcome. In this patient cohort, only one patient suspected to have prostate cancer with a PSAD ≤ 0.15 was found to have high-grade prostate cancer. This is consistent with prior studies that have shown that a PSAD threshold of 0.15 ng/mL/mL has increased sensitivity and specificity for the detection of clinically significant prostate cancer^(^^15^^)^. The use of this threshold could have avoided an unnecessary TRUS-guided biopsy in approximately half of the men in our sample with suspected prostate cancer.

In the setting of a negative mMRI, patients under AS were found to have a greater rate of low and intermediate to high-grade cancer than patients who were only suspected of having prostate cancer. This was expected, as untreated low-grade disease is at a risk of progression to higher grade disease. Furthermore, about two thirds of patients with prostate cancer have multifocal disease, which is not always detected at baseline and is sometimes characterized by high GS. This result supports our initial hypothesis that the wide variation in reported NPVs of mpMRI is, at least to some extent, a result of differences in baseline prevalence of high-grade prostate cancer in the populations that were investigated.

Our study has limitations. It was performed at a single tertiary care institution. Our results may not be universally applicable as our institution performs a high volume of prostate mpMRI examinations than most imaging centers and therefore our radiologists are more adept at interpreting such studies and using the PIRADS grading system. The use of an endorectal coil has become less common and that may also limit the generalizability of our results. Systematic TRUS-guided biopsies may miss cancers, but we should not expect this to be more or less common in men with suspected cancer versus those under AS. Despite obtaining significant results, the sample size of our study limits the precision of our estimates. Also, we only followed patients up to a single biopsy after mpMRI. It is conceivable that repeat biopsies would have detected additional high-grade disease.

In conclusion, the NPV of mpMRI for GS ≥ 3+4 is significantly higher in men with suspected prostate cancer than in men under AS. PSAD ≤ 0.15 further improved the prediction of intermediate to high-grade disease in patients without known cancer and it may be possible to avoid a TRUS-guided biopsy and its associated adverse events including the potential overtreatment of indolent tumors.
